# Supplementary figures and images for: Mycobacteria Tolerate Carbon Monoxide by Remodeling Their Respiratory Chain
Source: mSystems. 2021 May 11;6(3):e01292-20. doi: 10.1128/mSystems.01292-20 (PMC8125079; doi:10.1128/mSystems.01292-20)

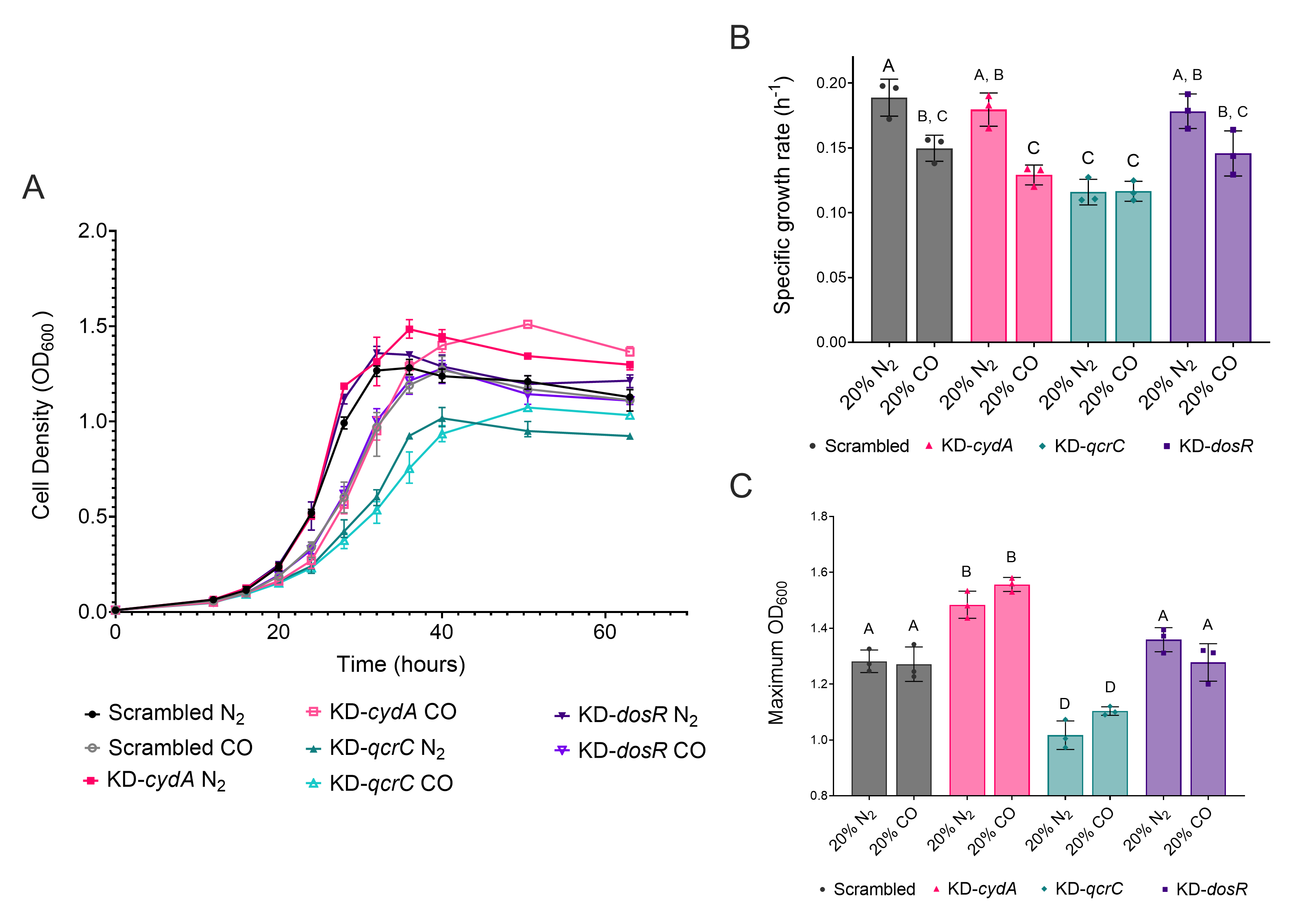

Supplement: FIG S1 [file mSystems.01292-20-sf001.tif]

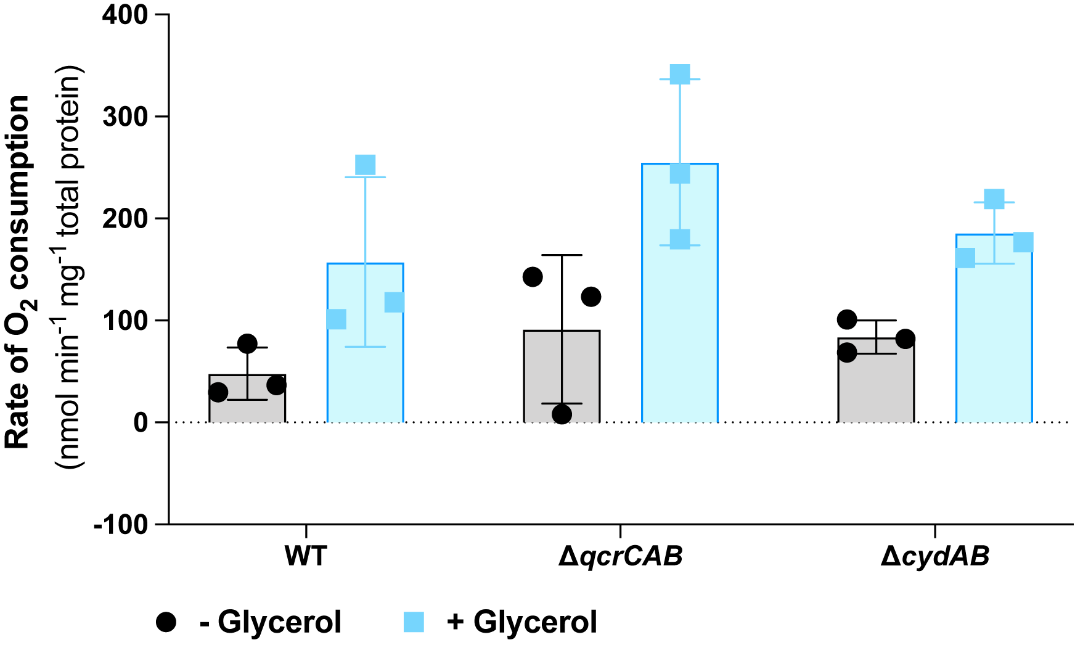

Supplement: FIG S2 [file mSystems.01292-20-sf002.tif]

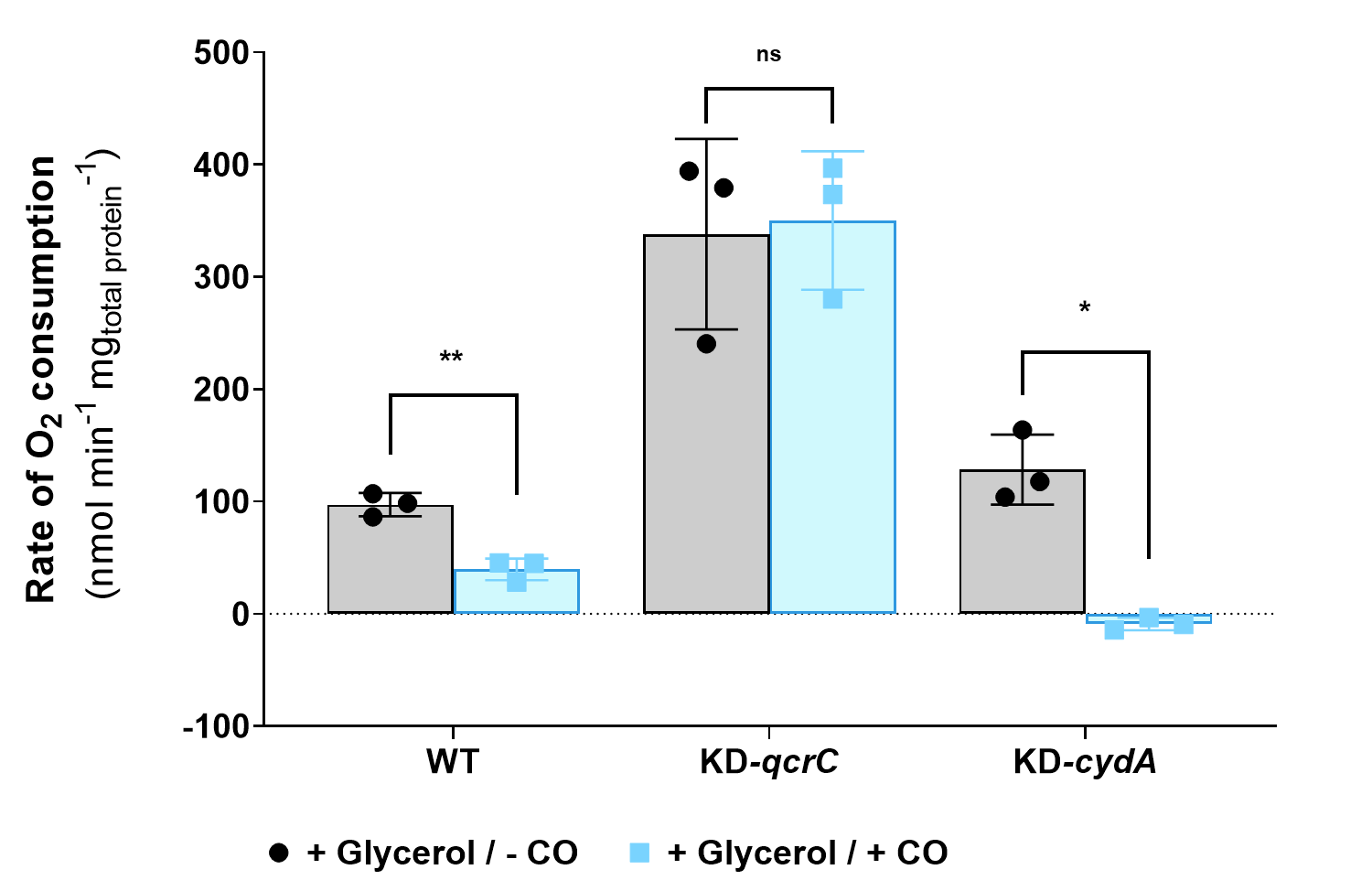

Supplement: FIG S3 [file mSystems.01292-20-sf003.tif]

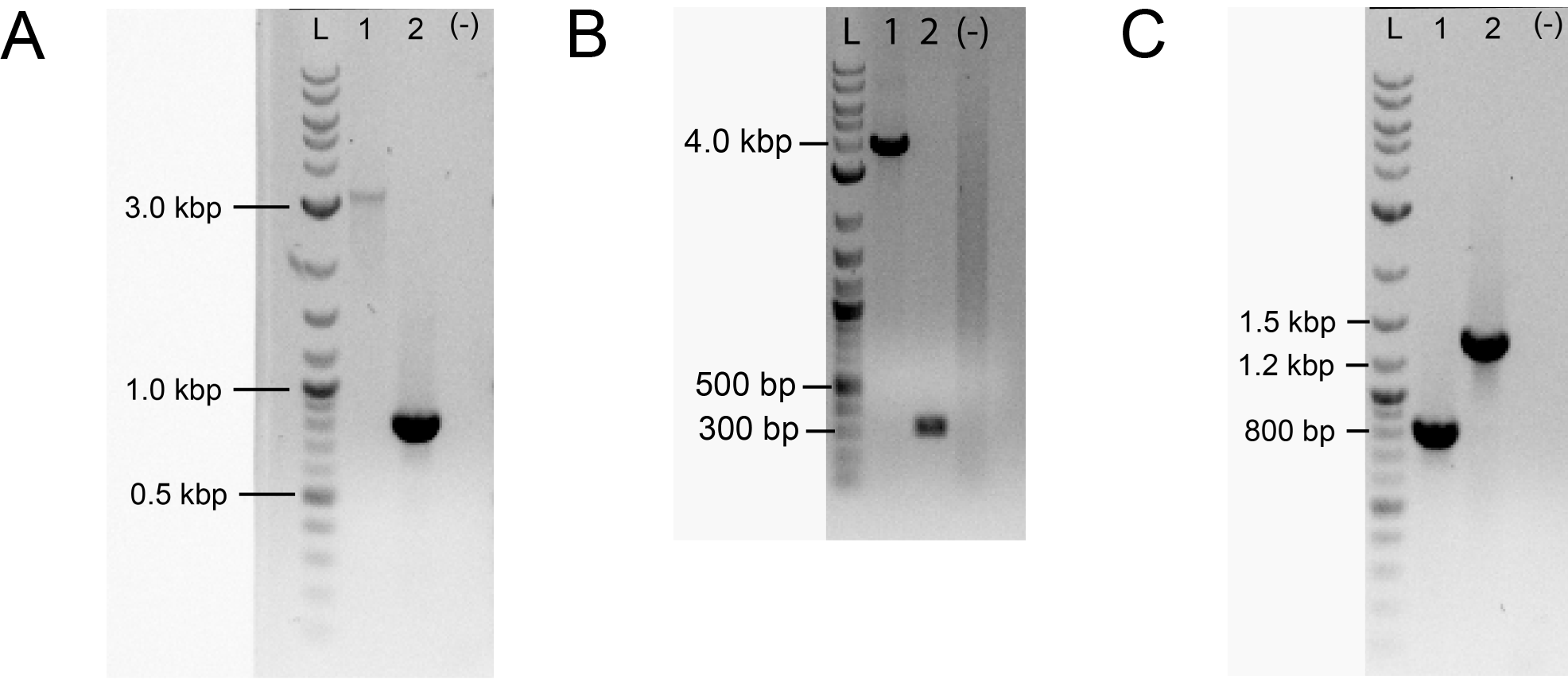

Supplement: FIG S4 [file mSystems.01292-20-sf004.tif]
