# Supplementary material for: Mycobacteria Tolerate Carbon Monoxide by Remodeling Their Respiratory Chain
Source: mSystems. 2021 May 11;6(3):e01292-20. doi: 10.1128/mSystems.01292-20 (PMC8125079; doi:10.1128/mSystems.01292-20)
Supplement: TABLE S2 [file mSystems.01292-20-st002.docx]

| **Strain** | **Description** | **Source/citation** |
| --- | --- | --- |
| mc^2^155 | Wild-type *M. smegmatis* | (41) |
| *ΔcydAB* | Clean deletion of the *cydAB* genes, mc^2^155 background | Prof. Greg Cook, Otago University (33) |
| *ΔqcrCAB* | Clean deletion of the *qcrCAB* genes, mc^2^155 background | Prof. Greg Cook, Otago University (33) |
| *ΔdosR* | *dosR* knockout; 597 bp of *dosR* replaced with a hygromycin resistance cassette, mc^2^155 background | (17) |
| Scrambled | CRISPRi non-targeting control; mc^2^155 transformed with the integrating CRISPRi plasmid pLJR962 with non-targeting sgRNA | Plasmid from (44) |
| KD-*cydAB* | mc^2^155 transformed with pLJR962 with sgRNA targeting *cydAB* | This study |
| KD-*qcrCAB* | mc^2^155 transformed with pLJR962 with sgRNA targeting *qcrCAB* | This study |
| KD-*dosR* | mc^2^155 transformed with pLJR962 with sgRNA targeting *dosR* | This study |
