# Supplementary material for: Mycobacteria Tolerate Carbon Monoxide by Remodeling Their Respiratory Chain
Source: mSystems. 2021 May 11;6(3):e01292-20. doi: 10.1128/mSystems.01292-20 (PMC8125079; doi:10.1128/mSystems.01292-20)
Supplement: TABLE S3 [file mSystems.01292-20-st003.docx]

| **Primers** | | | |
| --- | --- | --- | --- |
| **Description** | | **Sequence** | |
| pLJR962 sgRNA sequencing | | TTCCTGTGAAGAGCCATTGATAATG | |
| pLJR962 SapI site fwd | | GCTCTTCAGGATCTGACCAGGGAAAATAGCCCTC | |
| pLJR962 SapI site rev | | GCTCTTCACTGAAAAAAATAAAAAAGGGGACCTCTA | |
| *ΔcydAB* screen fwd | | AGCACCATGAAGATCGGGAT | |
| *ΔcybAB* screen rev | | ATCGCTACTACACTCCGTCG | |
| *ΔqcrCAB* screen fwd | | TCGTCTCGTACTACTGGCAC | |
| *ΔqcrCAB* screen rev | | GTAGGGGAGTGGCGCATATA | |
| *ΔdosR* screen fwd | | CATGGATCTGACGCTATGGC | |
| *ΔdosR* screen rev | | GAACGTTTCAACCGGGCC | |
|  | | | |
| **sgRNAs** | | | |
| **Gene target** | **Target sequence within gene** | | **Double-stranded oligo cloned into pLJR962** |
| *cydA* | CTTCTTCTTCGAGTCCACGTTCATCGGGC | | AAACTTCGAGTCCACGTTCATCGGGC----  ----AAGCTCAGGTGCAAGTAGCCCGAGGG |
| *qcrC* | CTTCTGCTGCTGATCGGTCTGGCAGTC | | AAACTGCTGATCGGTCTGGCAGTC----  ----ACGACTAGCCAGACCGTCAGAGGG |
| *dosR* | TTTCTGGTCGACGACCACGAGGTGGTGC | | AAACTCGACGACCACGAGGTGGTGC----  ----AGCTGCTGGTGCTCCACCACGAGGG |
